# The St. Louis Medical Society

**Published:** 1857-11

**Authors:** L Ch. Boisliniere

**Affiliations:** Secretary


### SELECTIONS.

### *St. Louis Medical Society.*

DR. McGINTIE, President, in the Chair.

Reported by L Ch. Boisliniere, M. D., Secretary.

*On Carbuncles. Case of Empyema. Distinctive signs between Empyema and Hepatic Abscess—Connection of Abscesses of the Liver and Chronic Dysentery. A case of Lacetation of Substance of the Brain. A case of a very large Thrombus of the Vagina. Vaginal Heronia. A discussion on Sun-Stroke—its Pathology and Treatment. A case of Abscess of the Liver operated on. Surgical cases. A case of Pseudo-Placenta Prævia. A case of Hydatitis or Mole Pregnancy. A case of retained Placenta, &c., &c.*

At this meeting of the Society Dr. LINTON introduced the subject of

### CARBUNCLES.

A subject of interest he remarked, especially as to its treatment. He had seen many cases of carbuncles, and he every day became more of opinion that the less surgical interference with this affection, the better. He could he thought attribute the fatal termination of one case to the use of the knife. He was aware that the best surgical authorities were in favor of deep incisions. Except, perhaps in a few rare cases, he thought that they could be dispensed with; he succeeded generally very well with poultices and leaving the carbuncle open itself; to this he added a sustaining treatment with quinine ammonia. He lately had a very bad case, which presented a very large carbuncle between the shoulders; this patient did very well with the above method. This case was also complicated with pellagra—a rare disease in this climate, he would remark.

Dr POPE said, that he was partial to the incision; it limits the ravages of large carbuncles; the small ones could do well without the knife. He had seen cases that died, he thought, from the want of the incision—small ones will generally progress favorably—large ones will, by a timely incision, be prevented from burrowing. He, also, believed in cauterization of the surfaces; the cauterization would produce a favorable alterative effect on the surfaces and their secretions. He remembered the case of a boy who presented in the back a very large carbuncle, at least fifteen inches in diameter, which quite exposed the dorsal muscles. The incision here prevented the further dissecting of the muscles—and the case did well.

Dr. McPheeters had lately under his care an old man with a large carbuncle. The patient applied to him when the carbuncle was already in the second or sloughing stage,—it was a very ugly looking ulcer with ragged edges and discharging unhealthy matter; the bottom of that sore was filled with soft and pultaceous exudations. A strong wash of nitrate of silver, together with a sustaining plan, was used in this instance, and proved very beneficial, for the patient, in spite of his old age—he was eighty—promptly recovered. Had he seen this patient in the early stage of the affection, he would have used the knife. The seriousness of this disease arose, he thought, mainly from the malignity of its character,—hence the satisfactory results obtained by a sustaining plan of treatment. However he was of the opinion, that, in some cases, the knife, followed by caustics and poultices, would limit the disease.

Dr. Smith said, that there was a distinction to be established between the inflammatory and the non-inflammatory carbuncle. In the former, a primary incision, by relieving the tension of the parts, would certainly tend to arrest the development of the affection and its future destruction of tissues, in the same way as an early incision in cases of paronychia, by removing the deep seated tension of the parts affected, proved very grateful to the patient. An early incision is imperatively demanded in those cases of inflammatory carbuncles where the pain is so great as to deprive the patient of sleep. The inflammatory carbuncles, he thought, were of a low type, and could, under certain circumstances, progress favorably without the incision, the sloughing in these cases was at times very extensive, and a sustaining course was the one to be adopted. However, in no circumstance, did he think could the incision aggravate the case.

Dr. McPheeters then related a case of

### HEPATIC ABCESS CO-EXISTING WITH CHRONIC DIARRHŒA.

It was the case of a man who died of a chronic diarrhœa at the Marine Hospital. His liver presented two large abscesses containing about a pint of pus. Dr. Brokaw, assistant physician to the same hospital, had investigated with care the condition of the liver of patients dying with chronic diarrhœa and dysentery, and the result of this examination was, that out of twenty-four patients who died of the above named diseases, seventy presented abscesses in the liver. This he would remark, was a strong confirmation of the views of Dr. Budd on the same subject.

Dr. McP. had also seen lately a case of

EMPYEMA, a case very interesting as being an illustration of one of the very rare terminations of this affection. The patient, an adult, had in the spring come to the hospital with simptoms of chronic pleurisy and effusion in the pleural sac. He was treated with mercurials, blisters and iodine, and discharged comparatively relieved. He resumed his occupations for a while, but a few weeks after applied again for admission. The former symptoms had reappeared, and in addition to these, he had now a well-marked prominence over the region of the liver, and pain on the right hypochondriac region. A few days after he began daily to expectorate small quantities of pus and sanies. It was like the matter usually found in abscesses of the liver. Owing to the prominence over the liver, Dr. McPheeters thought that there existed here some enlargement of the right lobe of the lung, together with hepatic abscess communicating with that lobe. In a short time diarrhœa set in, and the patient died of hectics.

A post mortem examination was made, and in cutting through the diaphragm a very large quantity of pus made its escape—it amounted to perhaps half a gallon. Contrary to his expectation the liver was found intract. The diaphragm and the adjoining layer of the pleura had strongly adhered. These formed the inferior boundary of this purulent collection, which had by a sort of endosmosis permeated the tissue of the lung and found its way to the bronchii, through which portions of this pus was daily expectorated as above mentioned. Had not the dissection in this case been performed with a great deal of care, the adhesion between the diaphragm and the pleura, so strong and intimate were they, might have escaped attention, and the case been mistaken for one of abscess of the liver. Another source of fallacy would have been to attribute the purulent expectorations to an abscess of the lung, but this viscus not only did not present the vast excavations that the great amount of pus found would have necessarily formed, but manifested only slight signs of enlargement at its base. By eliminating this two-fold source of error, it became therefore certain, that in this case, it was question of one of those very rare occurrences where a collection of pus in the pleural sac will tend to empty itself through the substance of the lung and the bronchii. He would call the attention of the society to the peculiar mode of termination observed in this instance, as being certainly very rare, and as having been but seldom noticed before my writers on this subject.

Dr. Hammer remarked, that this was a case of encysted empyema, and that the fact of their opening into the substance of the lungs was not so rare as thought to be. This mode of termination depended greatly upon the time this collection of pus had existed. If it had existed for a long time, by a sort of maceration that portion of the lung which had been immersed in the pus would undergo a sort of erosion, which would allow the matter to penetrate into its substance. This is not a rare termination of pleural empyema.

He would further say, that the differential diagnosis between empyema and abscess of the liver presented many points interesting to the pathologist. The obscurity of the diagnosis in these cases could however often be lessened by observing the sputa of the patient under the microscope; for, if the expectorated pus came from an hepatic abscess it would be found to contain liver-cells either in a perfect or rudamentary state In the same manner as the presence of these cells could have served to prove the fact of an abscess in the liver, their absence justifies one in excluding from one's diagnosis the supposition of that kind of abscess, and in establishing the existence of an encysten empyema. This difference in diagnosis would lead also to a difference of treatment. In this case the paracenthesis of the thorax would be advisable and would often prove successful. He regretted very much, on account of the rarity of this case, that an examination of the ejected pus could not have been procured; for it is a deplorable fact that medicines given internally are of little or no service in the treatment of empyema.

Dr. Smith doubted very much the frequency of the above mentioned termination of empyemas. He had, however, often seen cases of hepatic abscesses finding their way through the diaphragm to the lungs and then rejected. He would infer from the presence of the pus in the sputa either a case of gangrene of the lungs or one of hepatic abscess. The natural termination most frequent in empyema, he thought, was by pointing towards the skin. That hepatic abscesses should more frequently empty themselves into the lungs was obvious from the nature of the pathologiical process going on during the progress of these abscesses. At first, the abscess is circumscribed, but as it proceeds the hepatic tissue becomes broken down by the state of subacute inflammation which has invaded the organ; gradually the pus works its way towards the convexity of the liver, and a vast quantity now exists pressing upwards on the diaphragm; by the effect ef pressure continued for some time, as well as by a sort of maceration, the pleurodiaphragmatic septum the abdomen from the thorax at last gives way and an eruption of pus is made into the cavity of the chest, and the bronchii, through which it is finally expectorated. Every one acknowledged the great agency of pressure as hastening ulceration and causing absorption. He had referred to this pathological process, as it shows that if wrongly interrupted it may lead to the confounding of a suppurated hepatic abscess with empyema. He repeated that he thought the evacuation of an empyema through the lungs certainly a very rare and exceptional occurrence, and difficult to explain except under the supposition of the coincidence of a gangrene of the lung or an abscess of that organ.

Dr. Hammer answered, that this termination was rare, but because cases of empyema were fortunately for mankind very rare. He admitted the effects claimed by Dr. Smith for pressure in producing absorption, but was pressure absent in an empyema? Besides, he saw no reason *a priori* why the pus of empyema should not enter the substance of the lungs: this seemed to him the most natural method; for the pus of an hepatic abscess, before reaching the lungs, has to traverse eight times the thickness of tissue that the pus of an empyema has to perforate before reaching the same organs. The substance of the lung by macerating in the pus of the empyema must also become more friable, a part of the corroding action that non-laudable pus under certain circumstances will sometimes acquire. How does nature cure an empyema? by three methods, said he; 1st, by absorption; 2dly, by pointing; 3dly, by openings into the substance of the lungs—and the fact that empyemas do occasionally point externally, corroborates the assertion he had already advanced, that this process having to be performed through many tissues, before reaching the skin, *a fortiori*, could the pus permeate the lungs, which are defended against its invasion only by comparatively looser cellular tissue, etc.

Dr. Smith then related to the Society an interesting case of

LACERATION OF THE BRAIN.

Was called a few nights ago to a man who, he was told, had fallen through the hatchway of a boat. This is all the history he could get of the case. When he saw the patient he found him in a state of profound coma, the pulse at 40, the pupils undilatable, in fact quite insensible to light. He was bleeding profusely from the left ear, and a slight wound could be noticed on the right brow. Dr. Smith, from the state of profound coma and the bleeding from the ear, diagnosticated this a case of fracture of the base of the cranium. He stated that in cases of this description the diagnosis was often very obscure and difficult to establish, especially when the circumstances preceding the injury were unknown. From the symptoms observed, it might also have been laceration of the substance of the brain (which it eventually proved to be). If the site of the injury received had been over the course of the internal meningeal artery, a rupture of that artery and subsequent extravasation of blood and symptoms of compression might legitimately have been suspected. A *post mortem* examination settled however these difficulties. There was found laceration of the substance of the brain, but no fracture of the cranium. From where did then the hemorrhage which escaped from the ears proceed? was it from the tympanum or the vessels of the brain? He thought that it was from the latter source.

Dr. Hammer remarked, that whenever blood was seen to issue from the ears we should suspect a fracture of the temporal bone. These fractures are often overlooked; they run in a horrizontal direction, and the dura matter is here so firmly adherent that it is often difficult to separate it from the bone. This fracture will therefore escape attention if not diligently sought for.

Dr. McPheeters recollected a case corroborating the last mentioned opinion; it was the case of a man who died after a fall from a horse; had not the spunge been freely used in washing the blood over the site of a fracture of the temporal bone, that fracture would have passed unnoticed.

Dr. Pollak presented to the Society a very interesting pathological specimen—it was a

PLACENTA RETAINED TWO WEEKS IN UTERO.

A four months' fœtes had been expelled two weeks before this occurrence. The discharged placenta presented several notable anomalies.—It was below the normal size, and was characterized by a degree of carnification, which at first sight might have caused it to be mistaken for a mole or some other morbid uterine product. However, by a careful observation of the direction of the muscular fibres, the nature of the areolar tissue and the distribution of the rudiments of the vascular arrangement so characteristic of the after-birth, the true nature of the expelled body could be ascertained. A microscopic examination demonstrated besides, without doubt, the presence in this body of a number of placental cells. The history of this case was as follows: At 9 A. M., Dr. Pollak was summoned to the patient and found her literally weltering in her blood; the whole surface of the bed being covered with large coagula. She was, as might have been expected, in a state bordering on collapse. This flooding had begun the night previous at 10 o'clock, and without any premonitory symptoms. On examination, he ascertained that the vagina was clodded with blood. On attempting to remove the coagula, he experienced some difficulty in detaching one which seemed to be firmly imbedded in the canal of the ostincæ; this being effected at last, the hemorrhage ceased at once. The uterus was in a contracted state.—Brandy, Aromat. Spt. of Ammonia being freely administered, she gradually rallied. On inspecting the last removed coagulum, the placenta was discovered. Mrs. K., the patient, was then told by Dr. Pollak that she must have had a miscarriage, and that this was the most probable cause of her hemorrhage. She was also requested to tell what had become of the fœtus. She promptly replied, that she had miscarried just two weeks since of a four months' fœtus, and that this accident had been attended with no untoward circumstances. The midwife had assured her that "all was right;" she had been perfectly well since and out of bed the last ten days, attending to her domestic duties, and that she hardly had any lochial discharge. She then exhibited the fœtus, which she had preserved in a jar of alcohol.

Dr. Pollak gave also to the Society an account of a case of

HYDATIDS.

This was a supposed case of placenta prævia. The patient in this case had gone nine months, enlarging all the time, and with all the ordinary signs of pregnancy, stoppage of catamenia, dark areola, morning sickness, etc., so that herself and friends thought this a *bona fide* case of pregnacy. When called to her, Dr. Pollak said she had severe uterine pains and flooded profusely. He could not however ascertain whether there had been any premonitory flooding at the seventh or eighth month. He examined per vaginum and felt a soft and elastic body protruding through the os uteri. He thought it was a case of placenta prævia, when with a severe uterine pain the patient suddenly passed a very large mass of hydatids, after which the flooding subsided. The quantity of these acephalocysts was so large as to fill two night-vessels. Dr. Pollak remarked, the diagnosis of this case had been surrounded with a great deal of obscurity; it did not, as usual, present a rapid enlargement of the uterus and a discharge of blood and serum. He was aware that these symptoms were frequently present in cases of mole or hydatid pregnancies, but they had been wanting in this instance.

Dr. Boisliniere reported to the Society a case of

THROMBUS OF THE VAGINA.

The patient a primipara, of luco-phlegmatic habits, with a normal pelvis, after an ordinary labor, ending with rather severe expulsive pains in the birth of a living child, was left comfortable, when, an hour and a half after, he was called to see her in haste. He found her in the greatest suffering, crying loudly and assuring those around her that this was ten times more severe than her labor pains. She complained then of a burning sensation in the vulva and a severe bearing down on the rectum, which gave her a pressing inclination to go to stool. She suggested that it was piles. This pain was so agonizing that she had no words to express it. In fact, she could hardly utter two words in succession. This appeared to him the more strange from the great fortitude she had shown during her labor. Dr. Boisliniere confessed that he fell here into the same error committed by Dr. McBride in a similar case, and that he took this at first to be one of those violent tenesmic pains that are experienced sometimes after labor, when the child's head has quickly and violently swept over the vaginorectal septum—a sort of severe spasm—and he ordered opium suppositories, and hot fomentations to the parts; the latter, as proved on trial, only aggravated the evil; the pain continuing to increase instead of abating. He made then a vaginal examination, which revealed to him the true nature of the complaint—it was a very large thrombus, beginning on the left side, ascending along side the pelvis as high up as the finger could reach, filling the whole of the vagina, so that the finger could with difficult only be introduced, and descending as low down as the left labium, where it was characterized by great hardness and elasticity. This tumor was rapidly enlarging—he could ascertain this by keeping the end of his finger on its surface—the patient was in the greatest agony, her pulse was getting quick, her countenance anxious and distressed. There was only one course to follow—Dr. Boisliniere, under these circumstances, availing himself of the great experience of Dr. M. M. Pallen in matters of this sort, sent for him, and it was decided to open the tumor largely at once. Dr. Pallen then incised the tumor which was projecting and bluish—his incision extended to three inches in length and reached the cavity of this thrombus, after which he emptied it of the coagula it contained, except the most adherent at the bottom for fear of inducing hemorrhage, and a sponge was introduced in the vagina to act as a tampon. The patient was immediately relieved and recovered well. The amount of clotted blood extracted must have been several ounces.

Dr. Pallen said, that the case related by Dr. Boislinere was a very interesting one ; interesting on account of its rareness, and on account of the fatality often attending them. He believed that the first published case in British midwifery, was that of Dr. McBride, which occurred in 1776. It had however been observed before. Since then, it has been noticed and described by various writers. Dr. Denman saw three cases, and all recovering, he was induced to think that the disease was devoid of danger; subsequent experience has, however, shown that he was mistaken—death has frequently occurred.

This disease, which consists of an effusion of blood into the areolar tissue, may be in one or both labia, may extend into the pelvis, and into the perineum. It doubtless, sometimes, occurs in the unimpregnated female; but it is usually an accompaniment of labor, appearing either during its progress, or soon after. The swelling of the labia, and the feeling of weight are succeeded by great pain. If the distension be very great, the pain is agonizing—fever of an active kind ensues, and even delirium. The patient can only lie on her back, with her thighs widely separated. The tumor is of a lived color, almost black, and is extremely tender.

Dr. PALLEN added, that there could be no doubt that the effusion arose from rupture of some vessel. In a state of pregnancy, the vessels are in a varicose condition, and their rupture easily produces a large discharge of blood. What particular blood-vessel is ruptured, is not determined, probably the pudic vein.

If the tumor, said Dr. P., occurs before delivery, and increases slowly, and does not interfere with delivery, it might be trusted to nature. If it increase rapidly and interfere with labor, it ought to be laid open, and the coagula turned out. The same rules apply to the treatment, if it occur after delivery. If it be of slow formation, and not large, the application of cold cloths is all that is necessary; but if it form rapidly, and the pain is great, then we ought to cut it open, turn out the coagula, leaving those which are entangled in the areolar tissue, as they will tend to prevent further bleeding. This being done, the tampon should be used, to produce pressure on the blood-vessels.

Dr. BOISLINIERE said, that a remarkable circumstance attending this case was the rapid formation of the thrombus one hour and a half after the birth of the child. This, said he, could only be explained on the supposition that this bloody tumor had begun to form before delivery, and that the head of the child had acted as a tampon, favoring the formation of coagula over the mouths of the bleeding vessels, thus arresting the hemorrhage for a time, and that these coagula having given way only an hour and a half after the expulsion of the child, then the hemorrhage began again to fill the tumor. He would also add, that the view of Dr. Pallen, that these hemorrhages depended upon a varicose condition of the veins of the pelvis, is confirmed by the fact established by statistics, that thrombus may occur indifferently after severe and protracted labors, or instrumental labors, as well as after easy and short labors, and that the occurrence of these tumors arose mostly from the general varicose diathesis which characterizes the state of utero-gestation especially with certain women of lymphatic temperament. Hence that fact that as this state of varcosity is more frequent with women who had several children, so should cases of thrombus be mostly found among these.

Dr. Pallen remarked also, that a careless observation might cause a thrombus to be mistaken for a vaginal hernia, but surgeons had established rules sufficiently explicit to guard against this error.

Dr. Gregory said, that the diagnosis of tumors about the vagina was sometimes surrounded with some difficulties; he had himself seen a case diversely diagnosticated as a vaginal hernia and an encysted tumor, which proved to be the former. These thrombi might at first sight be confounded with the different tumors which make their appearance in the vagina, whether their point of departure be from the cellular investments of the interior of this canal, or from some morbid enlargement of the ovaries or even a vesical calculus. Vaginal rectoceles or cystoceles might also be mistaken for a thrombus. However, the history of the case will be here of the greatest assistance to enlighten the diagnosis. Moreover, the form, the consistency, the situation of this tumor, its sensibility to pressure, the suddenness and rapidity of its formation, must be taken into consideration as well as its color when this can be appreciated. To distinguish this from a vesical calculus or a vaginal cystocele, it must be remembered that a thrombus has very seldom its seat at the anterior portion of the vagina. The facility of penetrating with the finger into the sac of a vaginal rectocele, will easily enable one to eliminate this from the diagnosis.

Dr Pallen related a case of pleurisy, terminating in serious and purulent effusion, and asked the opinion of the members present as to the

ABSORBABILITY OF PUS.

Dr. Gregory, in reply to this question, said, that he would remark, that all pathologists agreed that pus was absorbable; but that when it existed in considerable quantity the surgical rule was now, as it has always been, to evacuate, provided it can be reached without too much danger to the involved parts. The absorption of pus from the eye has been observed frequently, and doubtless deep collections beyond our observation are occasionally thus disposed of. The case of empyema, cited by Dr. Pallen, may have been originally hydrothorax, the lymph corpuscles diffused in the serum occupying the pleural sac degenerating into pus at a subsequent period. Vogel mentions a remarkable case in point.—He said, added Dr. G., that the fluid discharged by the two first operations of paracentesis performed on a patient, contained fibrin in solution, and in a short time coagulated. On the third occasion, it no longer contained fibrin, but, on the other hand, pus corpuscles. A few days after the last operation the patient died. On dissection it was found that the pleural cavity was completely invested with a thick pseudo-membrane, which was already half organized, and must therefore at all events have existed several days previous to death. The pus could therefore only be formed in the fluid; but since the fluid which was first discharged contained fibrin, it is clear that this constituent was not originally absent, but was doubtless consumed in the formation of pus. Nay, more, said Dr. Gregory, the incipient formation of pus has been observed in plasma entirely separated from the body. For instance, Dr. Helbert took plasma from beneath the cuticle raised by an ordinary blistering plaster; it exhibited no corpuscles of any kind; after standing for six hours, minute corpuscles were formed exactly analogous to those which appear in wounds when the formation of pus commences. Repeated experiments invariably gave the same results.

The absorption of pus, he believed, was generally a slow process; the corpuscles may remain a considerable time unchanged after the removal of the "liquor puris." In the course of time the pus cells wither, undergo the fatty or calcareous degeneration, or ultimately crumble into minute particles, liquify and re-enter the vascular current. The uncertainty of absorption in hydrothorax, the danger of purulent degeneration, after which resorption is much more doubtful and tardy, indicate the propriety of early paracentesis as practised for many years in this country by Dr. Bowditch, of Boston.

Dr. Pope remarked, that whereas the absorption of pus was rare occurrence in those cases, he had adopted the rule of performing early paracentesis. Three-fourths of the cases he had operated on had recovered.

Dr. Hammer could not let this opinion pass uncontroverted. For him, the absorption of pus was the rule. He had certainly seen it absorbed in cases of hypopyum.

Dr. Pope answered, that in the majority of these cases it was not pus but lymph that was present.

Dr. Hammer said, he had no doubt that in hypopion pus was present. A hypoyum, said he, was nothing but an accumulation of pus from the opening of an abscess of the cornea into the anterior chamber of the eye.

He had seen several cases in which this pus had evidently been absorbed.

Dr. Hammer said, that he had lately operated on a man for

STONE IN THE BLADDER.

The patient aged about thirty, of apparently good constitution, inclined to corpulency, presented however some indications of a strumous habit. For two years this man had presented all the rational signs of a calculus. Two physicians who examined him did not recognize its presence. The patient was in low spirits, suffering great pains, constant desire to urinate, which he could satisfy only by a frequent use of the catheter—at times he passed bloody and purulent urine. Being examined, the stone was soon recognized, it was lying on the neck of the bladder, and this was found much contracted. The presence of several calculi was besides revealed. After inhaling chloroform, the patient was operated on by the lateral incision; a large stone of the size of a hen's egg and three smaller ones about the size of pigeon eggs were removed. The largest one being of a friable nature was previously crushed before being removed. Dr. H. said, that in this case he would have preferred the lithontriptic method, the patient's perineum being very much contracted and narrowed by an abnormal approximation of the tuberosities of the ischium, and also because there existed masses of fatty and cellulor tissue thickening this region in an uncommon manner. He preferred however the knife on account of the great irritability of the bladder, pus and blood having been passed, and also on account of the unusual contraction of that viscus.—The stones were found to be of the triple-phosphate variety, and the patient was, said he, thus far doing well.

Dr. Lonergan reported a case of rare occurrence, viz:

A SECOND ATTACK OF CONFLUENT VARIOLA IN THE SAME SUBJECT.

The subject of this observation, a boy six years old, had been attended three years ago by the lamented Dr. Reyburn, during an attack of small-pox of a confluent character. The patient had not been vaccinated before. He fully recovered from this disease and enjoyed excellent health for three years, when he was again exposed to the contagion of small-pox which prevailed extensively in the neighborhood where he lived. In the beginning of this second attack the symptoms did not depart from those which are usually found. The eruption, however, gradually became general and confluent. In the course of a week typhoid symptoms made their appearance, the tongue became dry, low delirium set in, and the vesicles ceased to fill up, thirty-six hours before death, which occurred on the 10th day of the invasion. Dr. Lonergan remarked, that he had reported this case on account of the very unusual fact of *confluent* small-pox occuring twice in the same individual. He was aware that relapses of ordinary small-pox had been occasionally observed, although this had been strongly denied by very high authorities; but relapses from confluent variola were certainly very rare. A second attack of small-pox has occasionally been found much more severe and oftener fatal than the first; and patients who had hardly been marked by a first, said he, sometimes completely disfigured by a second attack. But these were exceptional facts, and generally, when a person has had small-pox once, the second attack will be much milder in its form, and present the characters of varioloid or even of varicella only.

In the management of those cases which assume a typhoid character from the onset, he found that the most appropriate plan of treatment was to sustain the patient with nourishing broths, occasionally with stimulants, and hardly giving any medicine except opiates at night; the latter remedy he found invaluable in controlling the delirium vigilans so frequent in this affection. He had during the winter and spring several cases, in which he had a fair opportunity of appreciating the value of this mode of treatment.

Dr. Pope gave the following interesting account of

A RARE CASE OF WOUND OF THE PALATE.

A child about four years old fell down stairs, having at the time a flageolet in her mouth. The sharp end of the instrument was driven against the palate, completely detaching it in a semicircular manner from the bones. The loose flap, attached only at the extremities, hung low down in the pharynx and was very much contused; a vertical laceration also existed but did not extend to the free margin of the palate; the voice was affected as in cases of lost palate. With much difficulty, by means of a porte-aiguille and curved needle, three sutures, one median and two lateral, were well placed, and the flap was thus held in situ. Silence and the use of liquids were enjoyed. On the second day, a well-marked eruption of scarlet fever took place, but very fortunately the throat entirely escaped. The wound meanwhile looked well until the fourth day, when, in consequence of a small slough, there was failure of union at the middle and left sutures. About one-third of the laceration was, however, firmly united, and this served to keep the flap sufficiently in place for granulation to go on and gradually close up the remaining space. This accident happened about three weeks ago, and now the whole wound is cicatrized without any abnormal opening, the line of cicatrix being very distinct, with slight irregularity in the middle at the junction of the soft to the hard palate. The voice is but little altered, and upon the subsidence of the stiffness of the palate, will, most likely, regain its usual tone.

This case is interesting from the admitted difficulty of performing the operation for velosynthesis in so young a subject.

